# MRI Findings in Neuroferritinopathy

**DOI:** 10.1155/2012/197438

**Published:** 2011-07-21

**Authors:** Emiko Ohta, Yoshihisa Takiyama

**Affiliations:** Department of Neurology, Interdisciplinary Graduate School of Medicine and Engineering, University of Yamanashi, 1110 Shimokato, Chuo, Yamanashi 409-3898, Japan

## Abstract

Neuroferritinopathy is a neurodegenerative disease which demonstrates brain iron accumulation caused by the mutations in the ferritin light chain gene. On brain MRI in neuroferritinopathy, iron deposits are observed as low-intensity areas on T2WI and as signal loss on T2∗WI. On T2WI, hyperintense abnormalities reflecting tissue edema and gliosis are also seen. Another characteristic finding is the presence of symmetrical cystic changes in the basal ganglia, which are seen in the advanced stages of this disorder. Atrophy is sometimes noted in the cerebellar and cerebral cortices. The variety in the MRI findings is specific to neuroferritinopathy. Based on observations of an excessive iron content in patients with chronic neurologic disorders, such as Parkinson disease and Alzheimer disease, the presence of excess iron is therefore recognized as a major risk factor for neurodegenerative diseases. The future development of multimodal and advanced MRI techniques is thus expected to play an important role in accurately measuring the brain iron content and thereby further elucidating the neurodegenerative process.

## 1. Introduction

Neuroferritinopathy is an autosomal dominant neurodegenerative disorder characterized by the deposition of iron and ferritin in the brain and a decreased level of serum ferritin. The disease is caused by a mutation in the ferritin light chain gene [1]. Seven different pathogenic mutations of the ferritin light chain gene have been identified [1–7]. These mutations are predicted to affect the tertiary structure and stability of the ferritin light chain polypeptide and may cause inappropriate iron release from ferritin polymers [8, 9]. It is supposed that the excess iron induces free toxic radical production, which leads to tissue oxidative stress and neuronal cell death [10–12]. The clinical features of neuroferritinopathy are characterized by the adult onset of extrapyramidal motor symptoms: dystonia, chorea, choreoathetosis, parkinsonism, and tremor. Some patients may present cerebellar ataxia, cognitive decline, and pyramidal signs [2, 3, 5–7]. The phenotypic signs of the disease are variable, even among members of the same family [1, 3]. Generally, there are no nonneurological symptoms [13], different from in other neurodegenerative brain iron accumulation diseases. The clinical features of neuroferritinopathy are not specific, and they overlap with those of common extrapyramidal disorders. It is difficult to diagnose neuroferritinopathy solely based on the clinical findings. Brain MR imaging in the disease is quite characteristic and it may facilitate differential diagnosis of neuroferritinopathy from other extrapyramidal disorders.

## 2. Brain MR Imaging in Neuroferritinopathy

We will review the findings in neuroferritinopathy with conventional MRI methods, T1-weighted imaging, T2-weighted imaging, and T2*-weighted imaging. On T1WI, there is a sharp contrast between the parenchyma and ventricles, and it is adequate for evaluating brain atrophy and cystic changes. T2WI is suitable for detecting the pathological processes with an increase in water content, such as gliosis, edema and axonal/neuronal loss, as hyperintense signals. On T2*WI with a gradient echo sequence, the signals are readily influenced by magnetic inhomogeneity. Therefore, T2*WI is sensitive enough to detect paramagnetism such as that of iron.

Signal abnormalities on brain MR imaging were observed in all affected individuals previously reported except for one case [13–15]. Despite the clinical differences, the neuroimaging is similar across cases [16]. The findings are usually bilateral and symmetric but sometimes asymmetric [3, 17]. Signal changes are found in widespread areas in the central nervous system [14]. 

 Radiological findings in patients with neuroferritinopathy have been shown to correlate with the observed pathology [18]. The abnormalities observed on MRI reflect four pathological changes: iron deposition, edema and gliosis, cystic changes, and cortical atrophy [1–3]. Each finding is described individually below.

### 2.1. Iron Deposition

Iron is essential for normal neuronal metabolism, but excessive iron may be harmful [19, 20]. It is known that iron overload can cause free-radical formation and neuronal damage. 

 Physiologically, brain iron appears to be found predominantly in the extrapyramidal system, in particular the globus pallidus, substantia nigra, red nucleus, and putamen. It has been shown that moderate levels of iron occur in the striatum, thalamus, cerebral cortex, cerebellar cortex, and deep white matter [21]. It is also known that iron deposition increases normally with age. The brain histopathology of affected individuals with neuroferritinopathy involves excess iron and ferritin deposits throughout the forebrain and cerebellum, notably in the basal ganglia [1–3]. The accumulation observed in affected patients exceeds that found in normal elderly individuals. However, these regions still exhibit the general distribution pattern for iron in the normal aging brain [1]. 

 On fast spin echo T2WI, iron deposits are demonstrated as low-intensity areas and as signal loss on gradient echo T2*WI [13, 22]. Comparison of T2WI and T2*WI sequences suggests that the T2* one is more sensitive for the detection of iron, while the T2 fast spin echo T2WI sequence is more frequently used in routine clinical practice [14]. In particular, the cortical iron deposition in neuroferritinopathy is hardly detectable on T2WI but is easily observed on T2*WI [14]. Generally, iron deposit regions are isointense on T1WI [23].

### 2.2. Degeneration

T2 hyperintense abnormalities are seen in the pallidum, putamen, caudate nucleus [1, 3], thalamus and dentate nucleus, and sometimes in the red nucleus and substantia nigra [16, 24] in patients with neuroferritinopathy. The border of a lesion has a tendency to be unclear and the signal is unequal. These changes are supposed to reflect tissue degeneration with edema and gliosis observed pathologically. Because of the increased water content, the lesions are detected as hyperintense signals on T2WI [25]. Around these hyperintense areas, hypointensity due to iron deposits is frequently seen.

### 2.3. Cystic Changes

On MRI in neuroferritinopathy, the bilateral cystic changes involving the pallidum and putamen are impressive. Cavities are demonstrated as low-intensity signals on T1WI and high-intensity signals on T2WI, compared with the CSF signal. In the region adjacent to a cystic lesion, severe loss of nerve cells and neuropil is observed pathologically. In one case, Vidal et al. reported that microcavities measuring up to 1.5 mm in diameter were seen in the putamen anatomically and that these cavities were consistent with small hypointense areas on T1WI and to hyperintense ones on T2WI on MRI [2]. This finding is thought to represent the beginning stage of cavity formation.

 McNeill et al. analyzed the MRI findings in 21 patients with neuroferritinopathy. In 52% (11/21 patients), they found that the globus pallidus and/or putamen coincided with a confluent area of hyperintensity and that this hyperintense area was likely to be due to fluid within an area of cystic degeneration. It is usually accompanied by a rim of peripheral hypointensity reflecting iron deposition. This is a characteristic imaging pattern in neuroferritinopathy. The presence of large cysts is thought to be a finding observed at an advanced stage [14].

### 2.4. Cortical Atrophy

On brain MRI in neuroferritinopathy, atrophy is sometimes noted in the cerebellar cortices and cerebral cortices, notably in the frontal lobe. Atrophy of the cerebellar and cerebral cortices has also been anatomically identified. Regarding on clinicoradiologic correlation, patients having cerebellar atrophy present ataxia [2, 3, 26], and ones having cerebral atrophy present cognitive decline [23, 26].

## 3. The Relationship between the Stage of the Disease and MRI Findings

The first MRI change is loss of the T2* signal due to iron deposits. In an early symptomatic stage, and even in an asymptomatic carrier, there is obvious signal loss on T2* imaging in the basal ganglia, especially in the globus pallidus, at considerable frequency. In conventional spin echo MR sequences, the signal change is inconspicuous and is observed as a minor low signal on T2WI [13]. There has only been one report of that brain MR T2WI was normal without evidence of iron deposition; however, it was obtained six years after the onset of neuroferritinopathy symptoms. In this case, the T2* sequence was not examined at that time. The follow-up MRI performed 16 years after the onset, however, showed typical abnormalities [15]. 

 With disease progression, the T2 hypointense signal and T2* signal loss become more pronounced [13]. The changes eventually extend to the thalamus, dentate nucleus, substantia nigra, red nucleus, and cerebral cortex. 

 In the middle stage of the disorder, T2 hyperintense abnormalities reflecting tissue edema and gliosis are observed. In the basal ganglia, this change is thought to represent precystic degeneration [13]. The hypersignal lesions are often intermixed with decreased intensity areas corresponding to iron deposits. The combination of hyperintense and hypointense abnormalities is found in the pallidum, putamen, thalamus, and dentate nucleus frequently and sometimes in the red nucleus and substantia nigra [17, 27].

 The characteristic finding on brain MRI at the advanced stage is symmetrical cystic degeneration of the basal ganglia [16, 28]. Pathologically, many microcavities due to the loss of neurophils and neurons are observed, which are consistent with hypointense areas on T1WI and with hyperintense ones on T2WI on MRI [2]. It is supposed that small cavities merge to form larger cavities with progression of the disease. The large cavities observed on MRI have been confirmed by macropathological investigation [1].

## 4. MRI Findings in Our Case

**Figure 1:**
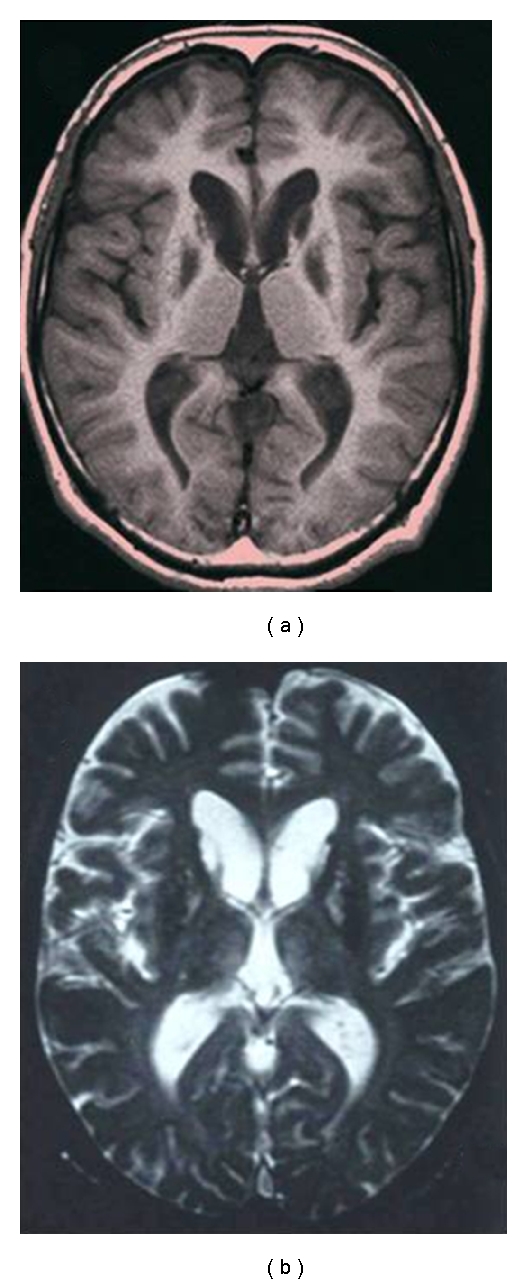
Axial section at the level of the basal ganglia in the patient at 35 years of age. (a) A T1-weighted image (TR 400 msec/ TE 14 msec) shows symmetrical hypointense signals in the head of the caudate nucleus and globus pallidus. (b) A T2-weighted image (TR 800 msec/TE 30 msec) shows hypointense changes in the lenticular nucleus. Hyperintense signals can be observed in the putamen and the head of the caudate nucleus.

**Figure 2:**
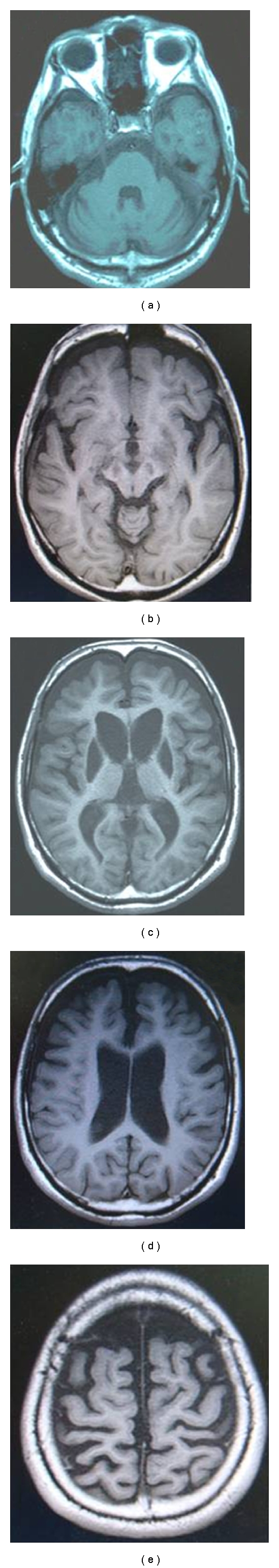
T1-weighted images (TR 400 msec/ TE 9 msec) of the same patient at 42 years of age. (a) A cross-section at the pontine level shows cerebellar cortex atrophy. (b) An image of a midbrain section demonstrates the hypointense change in the substantia nigra. (c) An image at the level of the basal ganglia shows symmetrical hypointense signals in the head of the caudate nucleus and globus pallidus. As compared with the findings at 35 years, the hypointense signals in the pallidum extend to the putamen. The cystic changes of the lenticular nuclei can be clearly observed. The shape of the cyst is fan shaped and exactly fits the region of the lenticular nucleus. The cerebral cortex in the frontal lobes is atrophic. The hypointense lesion in the caudate head observed in the image at 35 years of age seems to be combined with the hypointense signal of the anterior horn of the lateral ventricle. (d) In this image, enlargement of the lateral ventricles is evident. (e) This image shows cerebral cortical atrophy.

**Figure 3:**
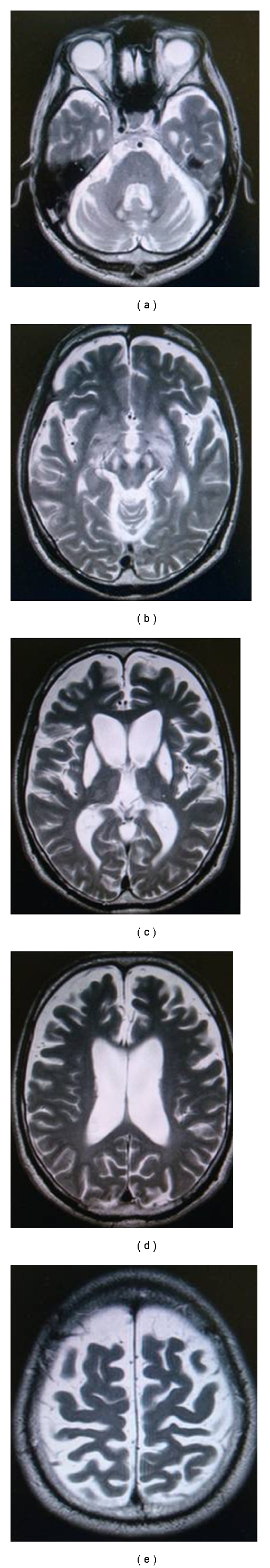
T2-weighted images (TR 3,440 msec/TE 89.4 msec) at 42 years old. (a) A cross-section at the pontine level exhibits bilateral and symmetrical signal loss with central hyperintense abnormalities in the dentate nucleus with cerebeller cortical atrophy. (b) An image of a midbrain section demonstrates the symmetrical increased signal intensity involving the substantia nigra. A decreased signal change is observed in the red nucleus. (c) An image at the level of the basal ganglia shows hyperintensity with a band of surrounding hypointensity affecting the putamen and pallidum. Foggy high signal changes can be seen in the inner part of the thalamus bilaterally. (d) An image of a section of the central part of the lateral ventricles. (e) An image of a section of the cerebral cortex. The signal change is not evident.

**Figure 4:**
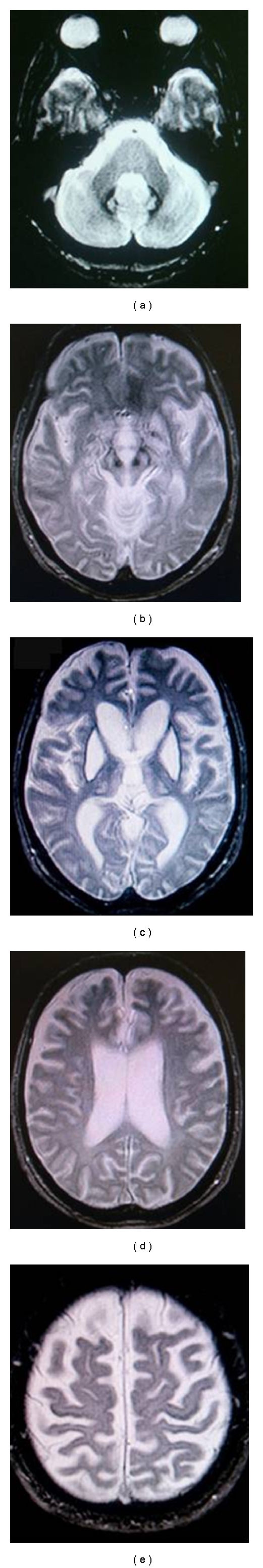
T2*-weighted images (TR 400 msec/ TE 25 msec) obtained with a gradient echo sequence in the same patient at 42 years old. (a) A cross-section at the pontine level. The signal loss with central hyperintense lesions in the dentate nucleus is more obvious than that observed on T2WI. (b) An image of the midbrain demonstrates the hypointense change in the red nucleus. (c) Cystic degeneration of the basal ganglia with a rim of peripheral signal loss is obvious. In the thalamus, bilateral hyperintense abnormalities surrounded by slight hypointensity can be seen. (d) An image of a section of the central part of the lateral ventricles. (e) The iron deposition in the cerebral cortex is detected as signal loss.

Brain MR images of our case are presented in Figures 1, 2, 3, and 4. Our patient is a 42-year-old Japanese man who first developed hand tremor in his middle teens. He noticed his right foot dragging at age 35, and generalized hypotonia, hyperextensibility, aphonia, micrographia, hyperreflexia, dystonia of his face, and cognitive impairment at age 42. Rigidity, spasticity, and chorea were not observed. His deceased mother had presented similar symptoms. His serum ferritin concentration was apparently low. He was tested by means of the molecular technique and diagnosed as having neuroferritinopathy because a mutation of the ferritin light chain gene was detected [5]. 

 All images presented here were taken with a 1.5 Tesla MR System. T1-weighted, T2-weighted, and T2*-weighted sequences were collected in the transverse plane. A T1-weighted image is useful for evaluating the atrophy and size of a cyst. As compared with the image at 35 years (Figure 1), that at 42 years demonstrates progression of the cystic formation and deterioration of the cortical atrophy in the frontal lobes (Figures 2(c) and 3(c)). Cortical atrophy can also be seen in the cerebellum (Figure 2(a)). Enlargement of the lateral ventricles is evident (Figure 2(d)).

 A T2-weighted image is valuable for detecting the combination of degenerative change and iron accumulation. A clear hyperintense lesion with a hypointense signal was found in the center of the dentate nucleus (Figure 3(a)). Foggy high signal changes were found in the inner part of the thalamus bilaterally (Figure 3(c)). These lesions are supposed to reflect the edema and gliosis observed pathologically. 

T2* images are valuable for detecting iron deposition. Iron deposits were indicated as signal loss in the dentate nuclei (Figure 4(a)), red nuclei (Figure 4(b)), thalamus (Figure 4(c)), at the periphery of the cysts (Figure 4(c)), and in the cerebral cortex (Figure 4(e)) in T2*-weighted images.

## 5. Differential Diagnosis

In this section, we provide an overview of the MRI findings in three other subtypes of neurodegeneration with brain iron accumulation (NBIA): pantothenate kinase-2 associated neurodegeneration (PKAN, formerly known as Hallervorden-Spatz syndrome), aceruloplasminemia, and infantile neuroaxonal dystrophy (INAD) for the differential diagnosis of iron deposition in the basal ganglia. Over the last decade, iron deposition in the adult brain is being increasingly recognized as an indicator of neurodegenerative processes in many chronic neurologic disorders including Parkinson disease and Alzheimer disease. We also mention the MRI findings in these common neurodegenerative diseases.

PKAN is a childhood-onset extrapyramidal disorder with aberrant iron metabolism caused by a mutation of the pantothenate kinase-2 (PANK2) gene [29]. Brian MRI findings in patients with the PANK2 mutation include hypointensity with an area of central hyperintensity in the globus pallidi on T2- and T2*-imaging, this characteristic sign being called the “eye-of-the-tiger” sign [14, 30–32]. McNeill et al. reported that two of 21 cases of neuroferritinopathy presented the “eye-of-the-tiger” sign and that the MRI findings in these iron accumulative disorders sometimes might overlap. He emphasized the importance of repeat imaging for a more accurate clinical diagnosis [14]. In the majority of PKAN cases, abnormalities are restricted to the globus pallidus and substantia nigra. In neuroferritinopathy, lesions in the globus pallidus, putamen, and dentate nuclei are consistently accompanied by ones in the caudate nuclei or thalami in a subset. The widespread location of lesions throughout the central nervous system is one of the characteristic MR findings in neuroferritinopathy patients [14].

 Aceruloplasminemia is an adult onset extrapyramidal disorder with iron deposition in the brain, liver, and reticuloendothelial system. It is caused by a mutation of the ceruloplasmin gene. The iron deposition in the central nervous system in aceruloplasminemia exhibits a distribution comparable to that in neuroferritinopathy, but in aceruloplasminemia, all basal ganglia nuclei and thalami are simultaneously involved as seen in T2-weighted and T2*-weighted images. A further distinguishing feature is the lack of the combination of hyperintense and hypointense abnormalities that is often observed in neuroferritinopathy. The low signal areas observed in aceruloplasminemia are homogenous. The cystic changes of the basal ganglia observed in neuroferritinopathy are rarely seen in aceruloplasminemia [13, 14, 33]. 

INAD is an autosomal recessive disorder with motor and mental deterioration, appearing within the first two years of life. It is due to mutations in *PLA2G6*. The characteristic MRI finding in INAD patients is cerebeller atrophy, which is often accompanied by signal hyperintensity in the diffuse cerebellar cortex [34]. In INAD, abnormal iron accumulation, detected as hypointense lesions on T2WI and T2*WI, is mainly observed in the globus pallidus, sometimes in the substantia nigra, and occasionally in the dentate nuclei. Even in advanced cases of INAD, there has been no report of iron accumulation in other structures [14]. It is different from the frequent involvement of the putamen, caudate and thalami in neuroferritinopathy. It has been observed in two INAD families with *PLA2G6* mutations that no iron accumulation was detectable on MRI despite severe clinical symptoms [35]. The cystic changes of the basal ganglia are not seen in INAD. 

Patients with Parkinson disease (PD) may show T2 hypointensity in many anatomic areas compared to normal controls including the substantia nigra pars compacta, dentate nucleus, subthalamic nucleus, and basal ganglia, probably reflecting an excess iron content. Quantitative studies have shown a 25% to 100% increase in substantia nigra iron levels in patients with PD compared to in normal controls. The correlation between T2 hypointensity of the substantia nigra and clinical severity has been demonstrated [22]. 

In patients with Alzheimer disease (AD), iron deposition in neurons, neurofibrillary tangles, and plaques has been reported pathologically. To detect brain iron accumulation using MRI in AD, investigators tried the high-solution 4.7 T MRI or field-dependent-rate-increase (FDRI) technique [22]. In these studies, increased iron levels were found in the basal ganglia. 

The signal change on MRI reflecting an excess iron content observed in patients with chronic neurologic disorders such as PD and AD is usually slight as compared with that in patients with NBIA including neuroferritinopathy. For exact measurement of brain iron or mineralization as a major risk factor for neurodegenerative diseases, multimodal and advanced MRI techniques are proposed [36, 37]. Improvement of MRI technique is one of the most important goals for correct diagnosis.

## 6. Conclusion

The variety of MRI findings including cystic degeneration of the basal ganglia, the combination of hyperintense and hypointense abnormalities, T2 hypointense lesions reflecting iron deposits, and cortical atrophy are specific to neuroferritinopathy. 

 In cases of suspected neuroferritinopathy, MRI may be useful for the detection and confirmation of such findings. At an early stage, since the abnormal iron deposits might be not detectable on T2-weighted imaging, T2*-weighted imaging is recommended. In most cases, there are clear distinguishing features for neuroferritinopathy and other iron accumulative disorders, including PKAN, INAD, and aceruloplasminemia. However, there is a degree of radiological overlap between neuroferritinopathy and these other iron accumulative disorders. 

The multimodal and advanced MRI techniques being developed to more sensitively and specifically quantify brain iron will be important for correct diagnosis and better understanding of the neurodegenerative processes in the pathological brain.

## References

[1] Curtis ARJ, Fey C, Morris CM (2001). Mutation in the gene encoding ferritin light polypeptide causes dominant adult-onset basal ganglia disease. *Nature Genetics*.

[2] Vidal R, Ghetti B, Takao M (2004). Intracellular ferritin accumulation in neural and extraneural tissue characterizes a neurodegenerative disease associated with a mutation in the ferritin light polypeptide gene. *Journal of Neuropathology and Experimental Neurology*.

[3] Mancuso M, Davidzon G, Kurlan RM (2005). Hereditary ferritinopathy: a novel mutation, its cellular pathology, and pathogenetic insights. *Journal of Neuropathology and Experimental Neurology*.

[4] Maciel P, Cruz VT, Constante M (2005). Neuroferritinopathy: missense mutation in FTL causing early-onset bilateral pallidal involvement. *Neurology*.

[5] Ohta E, Nagasaka T, Shindo K (2008). Neuroferritinopathy in a Japanese family with a duplication in the ferritin light chain gene. *Neurology*.

[6] Devos D, Tchofo PJ, Vuillaume I (2009). Clinical features and natural history of neuroferritinopathy caused by the 458dupA *FTL* mutation. *Brain*.

[7] Kubota A, Hida A, Ichikawa Y (2009). A novel ferritin light chain gene mutation in a Japanese family with neuroferritinopathy: description of clinical features and implications for genotype-phenotype correlations. *Movement Disorders*.

[8] Levi S, Cozzi A, Arosio P (2005). Neuroferritinopathy: a neurodegenerative disorder associated with L-ferritin mutation. *Best Practice and Research: Clinical Haematology*.

[9] Vidal R, Miravalle L, Gao X (2008). Expression of a mutant form of the ferritin light chain gene induces neurodegeneration and iron overload in transgenic mice. *Journal of Neuroscience*.

[10] Rouault TA (2001). Iron on the brain. *Nature Genetics*.

[11] Ke Y, Qian ZM (2003). Iron misregulation in the brain: a primary cause of neurodegenerative disorders. *Lancet Neurology*.

[12] Cozzi A, Rovelli E, Frizzale G (2010). Oxidative stress and cell death in cells expressing L-ferritin variants causing neuroferritinopathy. *Neurobiology of Disease*.

[13] Chinnery PF, Crompton DE, Birchall D (2007). Clinical features and natural history of neuroferritinopathy caused by the FTL1 460InsA mutation. *Brain*.

[14] McNeill A, Birchall D, Hayflick SJ (2008). T2* and FSE MRI distinguishes four subtypes of neurodegeneration with brain iron accumulation. *Neurology*.

[15] Mir P, Edwards MJ, Curtis ARJ, Bhatia KP, Quinn NP (2005). Adult-onset generalized dystonia due to a mutation in the neuroferritinopathy gene. *Movement Disorders*.

[16] Crompton DE, Chinnery PF, Bates D (2005). Spectrum of movement disorders in neuroferritinopathy. *Movement Disorders*.

[17] Caparros-Lefebvre D, Destée A, Petit H (1997). Late onset familial dystonia: could mitochondrial deficits induce a diffuse lesioning process of the whole basal ganglia system?. *Journal of Neurology, Neurosurgery and Psychiatry*.

[18] Crompton DE, Chinnery PF, Fey C (2002). Neuroferritinopathy: a window on the role of iron in neurodegeneration. *Blood Cells, Molecules and Diseases*.

[19] Harrison PM, Arosio P (1996). The ferritins: molecular properties, iron storage function and cellular regulation. *Biochimica et Biophysica Acta*.

[20] Olanow CW, Arendash GW (1994). Metals and free radicals in neurodegeneration. *Current Opinion in Neurology*.

[21] Morris CM, Candy JM, Oakley AE, Bloxham CA, Edwardson JA (1992). Histochemical distribution of non-haem iron in the human brain. *Acta Anatomica*.

[22] Stankiewicz J, Panter SS, Neema M, Arora A, Batt CE, Bakshi R (2007). Iron in chronic brain disorders: imaging and neurotherapeutic implications. *Neurotherapeutics*.

[23] Gregory A, Polster BJ, Hayflick SJ (2009). Clinical and genetic delineation of neurodegeneration with brain iron accumulation. *Journal of Medical Genetics*.

[24] Chinnery PF, Curtis ARJ, Fey C (2003). Neuroferritinopathy in a French family with late onset dominant dystonia. *Journal of Medical Genetics*.

[25] Ory-Magne F, Brefel-Courbon C, Payoux P (2009). Clinical phenotype and neuroimaging findings in a French family with hereditary ferritinopathy (FTL498-499InsTC). *Movement Disorders*.

[26] Ohta E, Nagasaka T, Shindo K (2009). Clinical features of neuroferritinopathy. *Clinical Neurology*.

[27] Wills AJ, Sawle GV, Guilbert PR, Curtis ARJ (2002). Palatal tremor and cognitive decline in neuroferritinopathy. *Journal of Neurology Neurosurgery and Psychiatry*.

[28] Burn J, Chinnery PF (2006). Neuroferritinopathy. *Seminars in Pediatric Neurology*.

[29] Zhou B, Westaway SK, Levinson B, Johnson MA, Gitschier J, Hayflick SJ (2001). A novel pantothenate kinase gene (PANK2) is defective in Hallervorden-Spatz syndrome. *Nature Genetics*.

[30] Hayflick SJ, Hartman M, Coryell J, Gitschier J, Rowley H (2006). Brain MRI in neurodegeneration with brain iron accumulation with and without *PANK2* mutations. *American Journal of Neuroradiology*.

[31] Hayflick SJ, Westaway SK, Levinson B (2003). Genetic, clinical, and radiographic delineation of Hallervorden-Spatz syndrome. *New England Journal of Medicine*.

[32] Sharma MC, Aggarwal N, Bihari M (2005). Hallervorden Spatz disease: MR and pathological findings of a rare case. *Neurology India*.

[33] Morita H, Ikeda S, Yamamoto K (1995). Hereditary ceruloplasmin deficiency with hemosiderosis: a clinicopathological study of a Japanese family. *Annals of Neurology*.

[34] Kóbor J, Javaid A, Omojola MF (2005). Cerebellar hypoperfusion in infantile neuroaxonal dystrophy. *Pediatric Neurology*.

[35] Paisan-Ruiz C, Bhatia KP, Li A (2009). Characterization of PLA2G6 as a locus for dystonia-parkinsonism. *Annals of Neurology*.

[36] Péran P, Hagberg G, Luccichenti G (2007). Voxel-based analysis of R2* maps in the healthy human brain. *Journal of Magnetic Resonance Imaging*.

[37] Cherubini A, Péran P, Caltagirone C, Sabatini U, Spalletta G (2009). Aging of subcortical nuclei: microstructural, mineralization and atrophy modifications measured in vivo using MRI. *Neuroimage*.

